# Predictors of Ventricular Abnormalities in Children with Idiopathic Ventricular Extrasystoles

**DOI:** 10.3390/children12020206

**Published:** 2025-02-09

**Authors:** Rita Kunigeliene, Odeta Kinciniene, Germanas Marinskis, Vytautas Usonis

**Affiliations:** 1 Clinic of Children’s Diseases, Institute of Clinical Medicine, Faculty of Medicine, Vilnius University, Universiteto St. 3, 01513 Vilnius, Lithuania; 2 Vilnius University Hospital Santaros Clinics, Santariskiu St. 2, 08661 Vilnius, Lithuania; 3 Clinic of Cardiac and Vascular Diseases, Institute of Clinical Medicine, Faculty of Medicine, Vilnius University, Universiteto St. 3, 01513 Vilnius, Lithuania

**Keywords:** ventricular extrasystoles, children, ventricular dysfunction, myocardial fibrosis, channelopathy, cardiomyopathy

## Abstract

*Background and Objectives*: Ventricular extrasystoles, which are the most common arrhythmias in healthy children and adolescents, could be a reliable factor for the prognosis of structural heart diseases. However, extrasystoles arising in hearts with primary myocardial diseases or channelopathies might cause life-threatening events or be associated with arrhythmia-induced cardiomyopathy. The relationship between ventricular extrasystoles and ventricular abnormalities in children remains controversial. The aim of this study was to evaluate prevalence of ventricular abnormalities in children with ventricular extrasystoles. *Materials and Methods*: This was a retrospective cohort study of pediatric outpatients in Vilnius University Hospital Santaros Clinics because of ventricular extrasystoles. The inclusion criteria were 3–18-year-old children with more than 5% extrasystoles per 24 h. The exclusion criteria were previous diagnoses of congenital heart defects, cardiomyopathies, and channelopathies. We reviewed the results of electrocardiography, cardiac imaging, and cardiogenetic tests. *Results:* In total, 131 patients (55.7% males) were included from a database of 915 patients, of whom 79.4% ventricular extrasystoles were found incidentally. Ventricular extrasystoles were monomorphic—95.4%, multiform—4.6%, and consecutive—29.8%. Cardiac magnetic resonance imaging was performed on 22.9% of patients with one-third of the pathological findings (ventricular dysfunction and myocardial fibrosis). Ventricular dysfunction was associated with a higher frequency of ventricular extrasystoles, with a median highest frequency of 26.5% per 24 h. Cardiogenetic testing was performed on only five (3.8%) patients, and RyR2 mutation was detected in one. *Conclusions:* According to our results, ventricular dysfunction was strongly associated with a higher burden of ventricular extrasystoles.

## 1. Introduction

Ventricular extrasystoles (VE) are a reliable factor for assessing the prognosis of structural heart diseases and survival in the adult population [1]. VE are the most common ventricular arrhythmias in healthy individuals [2]. Large population studies showed a prevalence of 0.51% of this arrhythmia in the general pediatric population [3].

VE can be found in structurally normal and abnormal hearts, and might be the first sign of underlying cardiomyopathy or channelopathy [4,5], or a cause of arrhythmia-induced cardiomyopathy [5].

However, most common pediatric VE are idiopathic [5]. Idiopathic VE are defined as those in patients without structural heart disease [6], and they have been regarded as benign in children [7]. Although VE in structurally normal hearts are considered benign, arrhythmia-induced cardiomyopathy has been widely described [5].

A correlation between VE burden and left-ventricular (LV) systolic dysfunction has been reported in adults [8]. Most pediatric studies have presented conflicting results regarding a direct correlation between VE burden and LV dysfunction [8]. Baman et al. discovered that a VE burden of more than 24% was associated with decreased LV systolic function [8]. In addition, recent data showed that an asymptomatic high burden of VE (34.7%; standard deviation: 6.3%) can also be associated with left-ventricular dysfunction in children [9]. High-load VE may represent a subclinical stage of cardiac dysfunction [10]. Long-term high-load VE have adverse effects on cardiac function, leading to an increased LV diastolic diameter, reduced ejection fraction, heart failure, and tachyarrhythmia-induced cardiomyopathy [10]. Differently from these results, Guerrier et al. did not report any significant relationships between the VE burden and LV systolic function in a cohort of <21-year-old patients [5]. Consequently, the relationship between VE and ventricular abnormalities in children remains controversial.

The aim of this study was to evaluate the prevalence of ventricular abnormalities or channelopathies in pediatric patients presenting with ventricular extrasystoles, and to assess the course of ventricular extrasystoles in pediatric patients with structurally normal hearts.

## 2. Materials and Methods

### 2.1. Study Population—Collection of Demographic and Clinical Data

We retrospectively reviewed the records of patients at the pediatric cardiology outpatient department of the tertiary care Vilnius University Hospital Santaros Clinics between 1st January 2015 and 30th December 2020. We used a depersonalized database of 3–18-year-old pediatric patients. All of the patients had been diagnosed with VE. This study was approved by the Regional Ethics Committee (no. 2021/10-1383˗859 (1)).

We included 3–18-year-old children with a maximum reach of ≥5% VE per 24 h. The exclusion criteria were diagnosed congenital heart defects, cardiomyopathies, channelopathies, and a lack of data in their medical documentation.

We gathered data from the electronic medical records and entered them into an anonymized spreadsheet. The demographic data of the patients were collected, including age and biological sex. The clinical data that were collected included symptoms (chest pain, palpitations, and dizziness/syncope) or being without any complaints (asymptomatic or VE discovered incidentally during routine check-up) and diagnostic test results (instrumental tests and cardiogenetic testing).

### 2.2. Cardiogenetic Testing

The results of cardiogenetic tests were collected from patients if performed. Cardiogenetic tests were performed only in patients with suspected channelopathies and/or cardiomyopathies [11].

### 2.3. Collection of Data from Electrocardiographic Studies

We evaluated all of the available 24-h Holter electrocardiography results (24-h-ECG). We assessed the total number of VE, the total percentage of VE in 24 h, VE characteristics such as monomorphic, multiform VE, and consecutive VE (repetitive VE of up to three consecutive beats), and ventricular tachycardia. We grouped the results of the 24-h-ECG as follows: initial 24-h-ECG (first 24-h-ECG results), maximal 24-h-ECG (the highest number of VE in 24-h-ECG of the patient), and the data of the last performed 24-h-ECG.

We also evaluated all of the available exercise stress test data. The exercise stress test ECG evaluated the relationship of VE with physical exertion and the presence of VE during warm-up, maximal exercise, and recovery from exercise phases; the reaction of the hemodynamic parameters; the presence of significant repolarization abnormalities (negative T-waves of ≥1 mm in depth in ≥2 precordial or inferior leads, and ≥0.1 mV ST-segment elevation); and a QTc of longer than 440 ms or shorter than 330 ms during the manual measurement and the calculation of the duration [12].

### 2.4. Collection of Data from Cardiac Imaging Studies

Routine transthoracic echocardiography was performed according to the ACC/AAP/AHA/ASE/HRS/SCAI/SCCT/SCMR/SOPE 2014 Appropriate Use Criteria for Initial Transthoracic Echocardiography in Outpatient Pediatric Cardiology [13]. The left-ventricular (LV) ejection fraction was assessed using the Teichholz formula. LV systolic dysfunction was defined as an LV ejection fraction of <55% or fractional shortening of ≤28% [7,14]. LV dilatation was defined by end-diastolic volumes or diameters of more than 2 standard derivations from normal according to the nomograms (*z*-scores of >2 standard deviations) corrected by the body surface area (BSA) and age, or BSA and sex [15].

CMR was performed if the patients met the indication criteria according to local protocols. The CMR imaging results were calculated with the BSA and compared according to age, sex, and BSA using *z*-score nomograms [16]. Ventricular dilatation was defined as an LV or RV end-diastolic diameter of more than two z-scores according to the nomograms [16]. Ventricular dysfunction was defined as when the left-ventricular ejection fraction was <55% and/or left- and/or right-ventricular dilatations were present [7,15,16,17,18]. Myocardial fibrosis (fibrotic foci) in CMR were visible during the late accumulation of contrast material in the myocardium [17,19].

### 2.5. Outcomes

At least one of the following listed indicators was defined as a primary outcome: (1) all-cause mortality; (2) life-threatening arrhythmias, such as sustained ventricular tachycardia or ventricular fibrillation; (3) documented development of ventricular dysfunction, myocardial fibrosis, or detected channelopathy or cardiomyopathy during cardiogenetic tests. The secondary outcome was documented as an increasing number of VE. For a better evaluation of the primary and secondary outcomes, we analyzed the data of existing patients’ follow-ups. We defined the follow-up dynamics of VE as disappeared (no VE detected in the last 24-h-ECG), decreased (decreased numbers of VE—more than 1% of maximal VE counts—in the last 24-h-ECG), stable (the same frequency of VE in the last 24-h-ECG), and increased (increased frequency of VE—more than 1% of maximal VE counts—in the last 24-h-ECG). We then evaluated the results of available imaging tests, cardiogenetic tests, electrocardiographic tests, and treatments, such as medical treatment or radiofrequency catheter ablation.

### 2.6. Statistical Analysis

R software (version 4.2.2) was used to perform the statistical analysis. We tested for a normal distribution of quantitative variables with the Shapiro–Francia test. Normally distributed variables were presented as means and standard deviations; non-normally distributed variables were presented as median, minimum, and maximum values; and categorical variables were presented as numbers (percentages). Comparisons of the quantitative variables between two groups were tested with Student’s *t*-test or a chi-square test according to the distribution. Comparisons of continuous variables between multiple groups were tested with a one-way analysis of variance (ANOVA) or the Kruskal–Wallis test according to the distribution. Comparisons of categorical variables were tested with the chi-square or Fisher’s exact tests. The correlation between variables was evaluated with Pearson’s correlation coefficient (r) or Spearman’s rank correlation coefficient (r_s_) according to the distribution. Univariable and multivariable logistic regression analyses were performed to evaluate the relationship between baseline covariates and the presence of myocardial abnormalities in CMR and genetic tests. A *p*-value of ≤0.05 was considered statistically significant. We used the Kaplan–Meier survival curve, created with the DATAtab software, to evaluate the natural history of VE.

## 3. Results

### 3.1. Study Population—Demographic and Clinical Data

**Figure 1:**
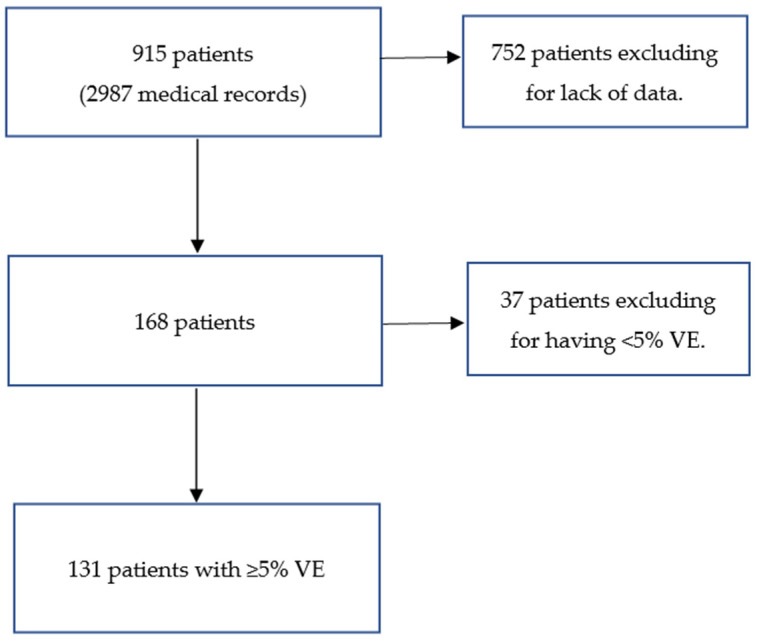
The study schematic of patient inclusion.

We screened 915 patients during the reference period (2987 medical records, from 1 to 85 records (median 13) of visits per patient) with a diagnosis code of I49.3 according to the International Classification of Diseases-10-AM. A schematic of patient inclusion in this study is shown in Figure 1.

A total of 131 patients met the inclusion criteria and not the exclusion criteria. The median age at the first visit was 11 (from 3 to 18) years old.

Table 1 presents the demographic and clinical characteristics of the patients.

A total of 27 (20.6%) symptomatic patients were included in our study, of which 12 patients presented with chest pain, 7 patients presented with syncope/presyncope, and 8 patients presented with palpitations. Generally, the symptoms were not dependent on biological sex (asymptomatic: 84.9% for males vs. 72.4% for females, *p* = 0.87; chest pain: 6.9% for males vs. 12.1% for females, *p* = 0.31; presyncope/syncope: 6.9% for males vs. 3.5% for females, *p* = 0.4). However, palpitations were more frequently experienced by females (12.1% vs. 1.4% (χ^2^ = 2343.5; *p* = 0.01)).

### 3.2. Cardiogenetic Testing

Cardiogenetic tests were performed on five patients (3.8%) only. The other four cardiogenetic tests did not reveal any clinically relevant mutations.

One asymptomatic 16-year-old girl with 5% multiform VE in the 24-h-ECG was determined to have a ryanodine receptor (RyR2) gene mutation (Case 1 in Table 2). RyR2 plays a major role in cardiac excitation–contraction coupling, and mutations in this isoform can give rise to cardiac arrhythmias [20]. However, we did not have complete history of the disease progression or any data of this patients’ CMR results to prove the diagnosis of cardiomyopathy.

### 3.3. Electrocardiographic Studies

All of the patients had 24-h-ECG results. The median count of VE in the initial 24-h-ECG was 10% (range: 0.7–47.2%). The patients reached their maximal VE count median of 12% (range: 5–48.6%) per 24 h.

The female sex was significantly associated with a higher number of VE. The median count of VE in the initial 24-h-ECG was 9% for males and 12% for females (χ^2^ = 2552; *p* = 0.04). The maximal 24-h-ECG VE median count was 9.8% for males vs. 15.7% for females (χ^2^ = 5837; *p* = 0.008). We found a strong positive correlation between the maximal VE count and the initial VE count (r_s_ = 0.83; *p* < 0.05) and no significant correlation between the initial or maximal VE count and the last VE count.We also did not find statistically significant differences between these VE morphologic characteristics and age (monomorphic vs. multiform *p* = 0.42), sex (monomorphic vs. multiform, *p* = 0.36; consecutive, *p* = 0.19), or the type of symptoms (consecutive, *p* = 0.56). However, consecutive VE were more common in older children (14 vs. 11 years, *p* = 0.001). In addition, monomorphic VE were more likely to be asymptomatic than multiform (89.03% vs. 69.2% (χ^2^ = 4.27; *p* = 0.04)).

The exercise stress test was performed on 57 (43.5%) patients. In 44 (71.2%) cases, the number of VE reduced during physical activity. Thirteen patients showed VE at maximal exertion. Eight patients had abnormal hemodynamic reactions to physical activity. However, no significant correlation between the hemodynamic reaction and VE counts was found. One patient demonstrated abnormal repolarization in the ECG during physical activity, and his CMR showed signs of myocardial fibrosis.

### 3.4. Cardiac Imaging Studies

Echocardiography was performed on 93 (70.1% of all) patients, and 96% had normal findings. The median LV ejection fraction was 63% (range: 50–73%). One patient showed a reduced left-ventricular ejection fraction, and three exhibited slight LV dilatation (with *z*-scores of up to two standard deviations). These results were confirmed during CMR.

Only 30 patients (22.9% of all) received CMR. In total, nine patients (Cases 2–10 in Table 2) demonstrated results defined as abnormal: six of them had signs of ventricular dysfunction and three displayed signs of myocardial fibrosis.

Table 3 presents the differences between normal and abnormal CMR results, and their relationships with the demographic data, clinical symptoms, and ECG characteristics according to the 24-h-ECG and during physical exertion and performed ablation.

Signs of ventricular dysfunction (LV systolic dysfunction/LV dilatation/RV dilatation) were demonstrated in six patients, two of which showed right-ventricular dilatation. These findings did not relate to sex (five boys (55.6%); χ^2^ = 60; *p* = 0.48) or age (χ^2^ = 66, *p* = 0.77). No patients experienced clinical symptoms. All of the patients had monomorphic and consecutive VE. Ventricular dysfunction was significantly related to the maximal VE counts over 24 h (median VE count: 26.5%; χ^2^ = 6.99; *p* = 0.008). Children with ventricular dysfunction more frequently underwent ablation.

Myocardial fibrosis was detected in three cases from our group: two boys and one girl. Only one patient experienced palpitations. All of the patients had monomorphic consecutive VE, and one patient demonstrated non-sustained ventricular tachycardia. The median VE count was 14% per 24 h.

Logistic regression was used to analyze the relationship between abnormal CMR and the initial VE count. The odds of abnormal CMR occurring increased by 12% (95% CI: [2–22%]) in the initial VE count (*p* = 0.04). In addition, ventricular dysfunction increased with an increase in the initial VE count by 19% (95% CI: [3–35%]) (*p* = 0.02). However, a logistic regression analysis of the association between pathological CMR/ventricular dysfunction and the initial VE count indicated no significant predisposition. There was also no significant association of VE progression with the initial and maximal VE counts (*p* > 0.05).

### 3.5. Follow-Up

**Figure 2:**
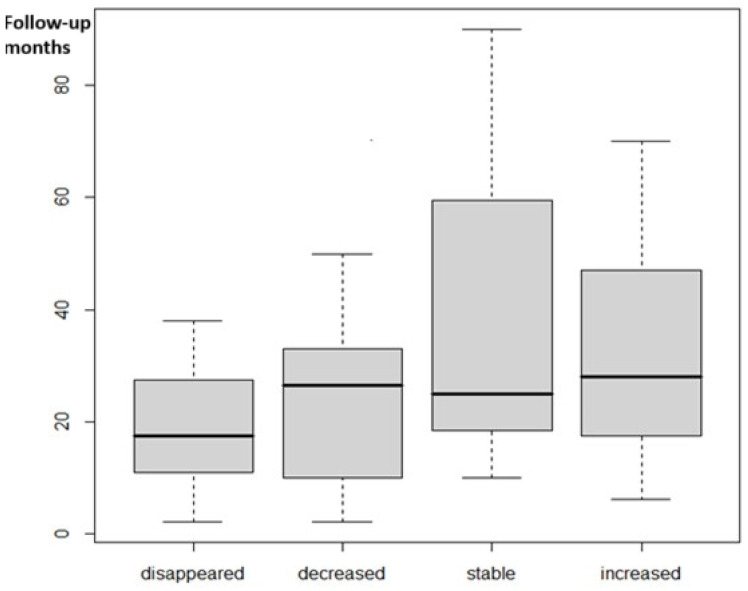
Median follow-up time in VE groups according to VE dynamics.

**Figure 3:**
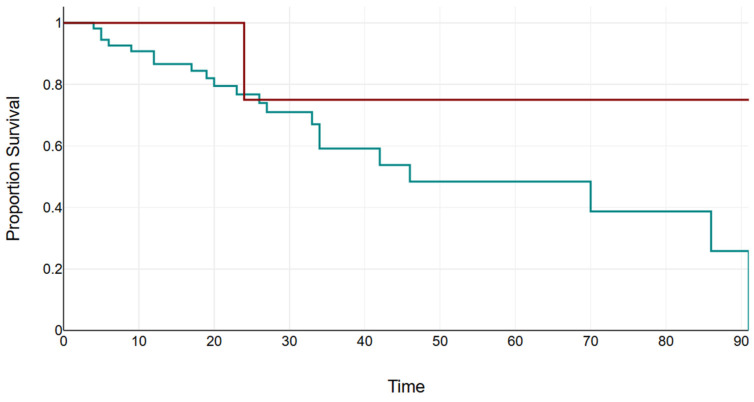
Kaplan–Meier curve estimate of the time of VE decrease. The *X*-axis is the time in months from the first visit with VE to the established increase in VE. The *Y*-axis is the cumulative proportion of patients remaining with VE. The red line represents patients with confirmed CMR abnormalities.

Follow-up data were available for 76 cases. The median duration of follow-up was 23.5 months (minimum: 3 months; maximum: 90 months). VE disappeared in 22 patients (28.9%), decreased in 37 patients (48.7%), were stable/without dynamics in 7 patients (9.2%), and increased in 8 patients (10.5%). The median duration of follow-up according to the VE dynamics did not differ between the outcome groups of the VE, as shown in Figure 2. The estimated time of VE decrease is shown in Figure 3.

No fatal cases or life-threatening arrhythmias occurred during the follow-up period.

## 4. Discussion

The most common type of pediatric ventricular arrhythmias are VE in both healthy children with structurally normal hearts and those with organic heart diseases [9]. The idiopathic VE were mostly benign in our study. The prevalence of ventricular abnormalities and abnormal cardiogenetic tests in our retrospective cohort study was only 7.7%, with one case associated with possible primary cardiomyopathy (with no data of CMR), six cases of ventricular dysfunction, and three cases of myocardial fibrosis.

VE are usually incidental electrocardiographic findings during outpatient follow-up [21]. VE were found accidentally in almost 80% of our patients. According to West et al.’s study, most (70%) patients with VE are asymptomatic [22]. Some children with VE may experience symptoms, but the severity of symptoms is not associated with the burden of VE [9]. Our study obtained the same finding. Although palpitations were associated with the female sex, we could not confirm an association between sex and VE morphologic characteristics or frequency.

Guerrier et al. reported no association between the VE complexity (monomorphic vs. multiform) and LV fractional shortening [23]. Monomorphic VE were mostly asymptomatic in our study. However, nine asymptomatic patients with monomorphic VE were diagnosed with myocardial abnormalities, and one patient with possible inherited cardiomyopathy (RyR2 mutation). All of the patients had monomorphic consecutive VE. Nevertheless, we did not prove statistically significant differences between myocardial abnormalities, VE characteristics, or demographic features in our patients. In our study, older children more frequently experienced consecutive VE, but we did not find an association between this VE characteristic and age.

Recent data have shown that a high burden of VE (greater than 20–30%) and asymptomatic ventricular tachycardia may be associated with left-ventricular dysfunction in children. Pediatric studies present confusing reports on the incidence of VE in arrhythmia-induced cardiomyopathy, ranging from 1.2 to 19.4%, and on the impact of VE burden [21]. Guerrier et al.’s study did not find a significant correlation between VE burden and LV dysfunction [23]. However, other studies have found a direct relationship between ectopic burden and ventricular dysfunction. Kakavand et al. reported four children with evidence of LV dysfunction, with an average ectopy burden of 36% [24]. Although the female sex was associated with a higher number of VE, there was no evidence of a burden of ventricular dysfunction in females. Our retrospective study found six patients with ventricular dysfunction, confirmed via CMR studies. All of these findings cannot be proved with inherited cardiomyopathy without performed cardiogenetic testing. Echocardiography confirmed only one case of ventricular dysfunction, and three cases of ventricular dilatation were borderline. Therefore, we believe that more detailed echocardiographic assessment should be used. Our study revealed that ventricular dysfunction was reliably associated with the maximum number of VE per 24 h. The initial 24-h-ECG counts of VE were not significant. Ventricular dysfunction occurred in those with a median maximal 24-h-ECG VE count of 26.5%. Because of the possibility of ventricular abnormalities, we believe that it is reasonable for patients with VE to have a periodic follow-up.

Cardiovascular magnetic resonance (CMR) imaging is an important non-invasive tool for assessing right- and left-ventricular dimensions and function [12,14,15,16], and for evaluating structural changes in the myocardium. Studies on the association between myocardial fibrosis and VE in the pediatric population are lacking. Our study revealed only three cases of myocardial fibrosis. Two cases exhibited fibrotic foci in the LV/RV junction. A study on young athletes by Crescenzi and co-authors showed an association between age and male predominance, and no association with symptoms and myocardial fibrotic changes in CMR [25]. Contrary to the possible hypothesis, the myocardial fibrosis group had lower numbers of VE. Our study showed lower VE numbers in the myocardial fibrosis group than in the ventricular dysfunction group, which may mean that VE are more likely a cause than a consequence of myocardial changes.

Recent studies have revealed that most cases of VE in children resolve spontaneously over time [21]. The median follow-up time of our study was 24 months, and VE completely resolved in 28.9% of patients. Our retrospective study could be the short follow-up period to develop significant dynamic results. Seventy-six percent of patients from our study were prescribed different antiarrhythmics, mostly beta-blockers, so the hypothesis of natural, benign development of idiopathic VE could not be fully proved.

Idiopathic VE and asymptomatic ventricular tachycardias are considered benign conditions with a good prognosis in the pediatric population [25]. However, some children are still at risk for fatal arrhythmias, such as ventricular paroxysmal tachycardia and/or ventricular fibrillation [25]. However, none of our patients experienced sudden life-threatening ventricular arrhythmia. Another limitation of our study was its retrospective nature, as we had limited data in the patients’ documentation. Therefore, we did not obtain comprehensive and standardized data, and the results may be susceptible to selection bias. In addition, our study was conducted at a single center with 131 patients, with a relatively small sample size. Furthermore, we had a limited number of patients who underwent CMR and cardiogenetic tests. Therefore, we had a limited number of cases with ventricular dysfunction, myocardial fibrosis, and inherited cardiac problems and could not adequately evaluate risk factors for arrhythmia-induced cardiomyopathy.

Our study’s main strength was the accurate assessment of VE in multiple 24-hour ECGs. This allowed us to accurately assess the initial and maximal VE per 24 h and evaluate the VE dynamics.

## 5. Conclusions

According to the results, ventricular extrasystoles in children were rarely (7.7%) associated with myocardial abnormalities or channelopathies. RyR2 mutation was found in one asymptomatic patient. We had three cases of myocardial fibrosis and six cases of ventricular dysfunction. However, ventricular dysfunction was strongly associated with a higher burden of extrasystoles. We believe that it is reasonable for patients with more than 26% VE per 24 h to have a more detailed assessment of ventricular function.

Further studies should focus on obtaining a better understanding of the relationships between ventricular dysfunction, evaluation using the assessment of myocardial deformation, myocardial fibrosis, channelopathies, and VE morphology, alongside relationships with stress tests. Delineating their connections may help to develop proposals for more effective diagnostic and prognostic tools.

## Figures and Tables

**Table 1:** Demographic and clinical characteristics of the study population.

Characteristic	n (%)
Sex:	
Male	73 (55.7%)
Asymptomatic	104 (79.4%)
Performed tests:	
24-h-ECG	131 (100%)
Echocardiography	93 (70.1%)
CMR	30 (22.9%)
Exercise stress test	57 (43.5%)
Cardiogenetic tests	5 (3.8%)
VE characteristics:	
Monomorphic	125 (95.4%)
Multiform	9 (4.6%)
Consecutive	39 (22.1%)
Ventricular tachycardia	1 (0.8%)
Life-threatening arrhythmias or fatal cases	0
Follow-up data	76 (58%)
Received medical treatment	100 (76.3%)
Radiofrequency catheter ablation	6 (4.6%)

**Table 2:** Characteristics of patients who had cardiogenetic abnormalities or CMR abnormalities.

Case N	CMR Abnormality	Genetic Test	Sex	Age	Symptoms	Initial 24-h-ECGVE, Count (%)	Maximal 24-h-ECGVE, Count (%)	Monomorphic VE	Consecutive VE	Exercise Stress Test	Follow-Up	Radiofrequency Catheter Ablation
1	ND	RyR2 mutation	F	16	None	ND	5%	+	-	ND	ND	ND
2	LV dilatation	ND	M	17	None	19,200 (18.8%)	19,200 (18.8%)	+	+	ND	ND	ND
3	LV dysfunction	ND	F	4	None	40,000 (35%)	60,383 (47%)	+	+	ND	26 months, decreased	RVOT
4	LV dilatation	ND	M	15	None	15,000 (13%)	15,000 (13%)	+	+	Recovery VE	24 months, stable	ND
5	RV dilatation	ND	M	7	None	20,345 (16.63%)	40,000 (35%)	+	+	ND	20 months, decreased	RV lateral wall
6	LV/RV dilatation	ND	F	13	None	24,000 (22%)	24,000 (22%)	+	+	Normal	43 months, decreased	ND
7	LV dilatation	ND	F	16	None	32,488 (31%)	32,488 (31%)	+	+	ND	24 months, decreased	RVOT
8	Fibrotic foci	ND	M	17	None	ND	20,790 (18.8%)	+	+	Recovery VE/abnormal repolarization	ND	ND
9	Fibrotic focus LV/RV junction	ND	M	15	None	2329 (1.9%)	6234 (5.63%)	+	+	Warm-up/maximal workload/recovery VE	23 months, decreased	ND
10	Fibrotic foci LV/RV junction	ND	F	17	Palpitations	15,860 (14%)	15,860 (14%)	+	+ tachycardia	ND	ND	ND

Abbreviations: F—female; LV—left ventricle; M—male; ND—no data; RV—right ventricle; RVOT—right ventricle outflow tract; “+” - yes; “-“ – no.

**Table 3:** Characteristics of patients with normal and abnormal CMR findings.

	Normal CMR (n-21)	Abnormal CMR (n-9)	*p*-Value
Sex			0.18
Male	6 (28.6%)	5 (55.6%)	
No symptoms	15 (71.4%)	8 (88.9%)	0.32
Chest pain	2 (9.5%)	0	
Palpitations	3 (14.3%)	1 (11.1%)	
Presyncope/syncope	1 (4.8%)	0	
Exercise stress test	n-11	n-3	
Warm-up VE	9 (81.8%)	3 (100%)	0.54
Recovery VE	9 (81.8%)	3 (100%)	0.54
Maximal-workload VE	4 (36.4%)	1 (33.3%)	0.45
Abnormal repolarization	0	1 (33.3%)	
VE characteristics			
Monomorphic	19 (90.5%)	9 (100%)	0.37
Multiform	2 (9.5%)	0	
Consecutive	8 (38.1%)	9 (100%)	0.17
Ventricular tachycardia	0	1	
24-hour-ECG			
Maximal VE:			
Median	12,379.5	20,790	0.02
%	11.43%	18.8%	
Initial VE results:			
Median	9710	19,772.5	0.06
%	8.2%	16.6%	
Radiofrequency catheter ablation	1 (4.8%)	3 (33.3%)	0.04

## Data Availability

On request due to data privacy, the corresponding author will provide the depersonalized datasets produced and/or analyzed during the current study.

## References

[1] Scorza R., Jonsson M., Friberg L., Rosenqvist M., Frykman V. (2023). Prognostic implication of premature ventricular contractions in patients without structural heart disease. Europace.

[2] Hoogendijk M.G., Géczy T., Yap S.C., Szili-Torok T. (2020). Pathophysiological Mechanisms of Premature Ventricular Complexes. Front. Physiol..

[3] Niwa K., Warita N., Sunami Y., Shimura A., Tateno S., Sugita K. (2004). Prevalence of arrhythmias and conduction disturbances in large population-based samples of children. Cardiol. Young.

[4] Sitorus G.D.S., Ragab A.A.Y., Houck C.A., Lanters E.A.H., Heida A., van Gastel V.E., Muskens A.J.Q.M., de Groot N.M.S. (2019). Ventricular Dysrhythmias During Long-Term Follow-Up in Patients with Inherited Cardiac Arrhythmia. Am. J. Cardiol..

[5] Cicenia M., Silvetti M.S., Drago F. (2021). When Should Premature Ventricular Contractions Be Considered as a Red Flag in Children with Cardiomyopathy?. J. Cardiovasc. Dev. Dis..

[6] Zeppenfeld K., Tfelt-Hansen J., de Riva M., Winkel B.G., Behr E.R., Blom N.A., Charron P., Corrado D., Dagres N., de Chillou C. (2022). 2022 ESC Guidelines for the management of patients with ventricular arrhythmias and the prevention of sudden cardiac death. Eur. Heart J..

[7] Spector Z.Z., Seslar S.P. (2016). Premature ventricular contraction-induced cardiomyopathy in children. Cardiol. Young.

[8] Cohen M.I. (2019). Frequent premature ventricular beats in healthy children: When to ignore and when to treat?. Curr. Opin. Cardiol..

[9] Bertels R.A., Kammeraad J.A.E., Zeelenberg A.M., Filippini L.H., Knobbe I., Kuipers I.M., Blom N.A. (2021). The Efficacy of Anti-Arrhythmic Drugs in Children With Idiopathic Frequent Symptomatic or Asymptomatic Premature Ventricular Complexes With or Without Asymptomatic Ventricular Tachycardia: A Retrospective Multi-Center Study. Pediatr. Cardiol..

[10] He Y.E., Xue Y.Z., Gharbal A., Qiu H.X., Zhang X.T., Wu R.Z., Wang Z.Q., Rong X., Chu M.P. (2021). Efficacy of radiofrequency catheter ablation for premature ventricular contractions in children. J. Interv. Card. Electrophysiol..

[11] Ackerman M.J., Priori S.G., Willems S., Berul C., Brugada R., Calkins H., Camm A.J., Ellinor P.T., Gollob M., Hamilton R. (2011). HRS/EHRA expert consensus statement on the state of genetic testing for the channelopathies and cardiomyopathies: This document was developed as a partnership between the Heart Rhythm Society (HRS) and the European Heart Rhythm Association (EHRA). Europace.

[12] D’Ascenzi F., Anselmi F., Adami P.E., Pelliccia A. (2020). Interpretation of T-wave inversion in physiological and pathological conditions: Current state and future perspectives. Clin. Cardiol..

[13] Campbell R., Douglas P., Eidem B., Lai W.W., Lopez L., Sachdeva R. (2014). ACC/AAP/AHA/ASE/HRS/SCAI/SCCT/SCMR/SOPE 2014 Appropriate Use Criteria for Initial Transthoracic Echocardiography in Outpatient Pediatric Cardiology: A Report of the American College of Cardiology Appropriate Use Criteria Task Force, American Academy of Pediatrics, American Heart Association, American Society of Echocardiography, Heart Rhythm Society, Society for Cardiovascular Angiography and Interventions, Society of Cardiovascular Computed Tomography, Society for Cardiovascular Magnetic Resonance, and Society of Pediatric Echocardiography. JACC.

[14] Tissot C., Singh Y., Sekarski N. (2018). Echocardiographic Evaluation of Ventricular Function-For the Neonatologist and Pediatric Intensivist. Front. Pediatr..

[15] Pinto Y.M., Elliott P.M., Arbustini E., Adler Y., Anastasakis A., Böhm M., Duboc D., Gimeno J., de Groote P., Imazio M. (2016). Proposal for a revised definition of dilated cardiomyopathy, hypokinetic non-dilated cardiomyopathy, and its implications for clinical practice: A position statement of the ESC working group on myocardial and pericardial diseases. Eur. Heart J..

[16] van der Ven J.P.G., Sadighy Z., Valsangiacomo Buechel E.R., Sarikouch S., Robbers-Visser D., Kellenberger C.J., Kaiser T., Beerbaum P., Boersma E., Helbing W.A. (2020). Multicentre reference values for cardiac magnetic resonance imaging derived ventricular size and function for children aged 0–18 years. Eur. Heart J. Cardiovasc. Imaging.

[17] Fogel M.A., Anwar S., Broberg C., Browne L., Chung T., Johnson T., Muthurangu V., Taylor M., Valsangiacomo-Buechel E., Wilhelm C. (2022). Society for Cardiovascular Magnetic Resonance/European Society of Cardiovascular Imaging/American Society of Echocardiography/Society for Pediatric Radiology/North American Society for Cardiovascular Imaging Guidelines for the Use of Cardiac Magnetic Resonance in Pediatric Congenital and Acquired Heart Disease: Endorsed by The American Heart Association. Circ. Cardiovasc. Imaging.

[18] DiLorenzo M.P., Farooqi K.M., Shah A.M., Channing A., Harrington J.K., Connors T.J., Martirosyan K., Krishnan U.S., Ferris A., Weller R.J. (2023). Ventricular function and tissue characterization by cardiac magnetic resonance imaging following hospitalization for multisystem inflammatory syndrome in children: A prospective study. Pediatr. Radiol..

[19] Tian J., An X., Niu L. (2017). Myocardial fibrosis in congenital and pediatric heart disease. Exp. Ther. Med..

[20] Van Petegem F. (2012). Ryanodine Receptors: Structure and Function. J. Biol. Chem..

[21] Bansal N., Mercadante A., Rochelson E., Mahgerefteh J., Clark B.C. (2020). Speckle Tracking Echocardiography in Pediatric Patients with Premature Ventricular Contractions. Pediatr. Cardiol..

[22] West L., Beerman L., Arora G. (2015). Ventricular ectopy in children without known heart disease. J. Pediatr..

[23] Guerrier K., Anderson J.B., Czosek R.J., Mays W.A., Statile C., Knilans T.K., Spar D.S. (2015). Usefulness of ventricular premature complexes in asymptomatic patients ≤21 years as predictors of poor left ventricular function. Am. J. Cardiol..

[24] Kakavand B., Ballard H.O., Disessa T.G. (2010). Frequent ventricular premature beats in children with a structurally normal heart: A cause for reversible left ventricular dysfunction?. Pediatr. Cardiol..

[25] Crescenzi C., Zorzi A., Vessella T., Martino A., Panattoni G., Cipriani A., De Lazzari M., Perazzolo Marra M., Fusco A., Sciarra L. (2021). Predictors of Left Ventricular Scar Using Cardiac Magnetic Resonance in Athletes With Apparently Idiopathic Ventricular Arrhythmias. J. Am. Heart Assoc..

